# Ecological Changes in Coyotes (*Canis latrans*) in Response to the Ice Age Megafaunal Extinctions

**DOI:** 10.1371/journal.pone.0116041

**Published:** 2014-12-31

**Authors:** Julie A. Meachen, Adrianna C. Janowicz, Jori E. Avery, Rudyard W. Sadleir

**Affiliations:** 1 Des Moines University, Anatomy Department, Des Moines, Iowa, United States of America; 2 Saint Xavier University, Department of Biological Sciences, Chicago, Illinois, United States of America; University of Queensland, Australia

## Abstract

Coyotes (*Canis latrans*) are an important species in human-inhabited areas. They control pests and are the apex predators in many ecosystems. Because of their importance it is imperative to understand how environmental change will affect this species. The end of the Pleistocene Ice Age brought with it many ecological changes for coyotes and here we statistically determine the changes that occurred in coyotes, when these changes occurred, and what the ecological consequences were of these changes. We examined the mandibles of three coyote populations: Pleistocene Rancho La Brean (13–29 Ka), earliest Holocene Rancho La Brean (8–10 Ka), and Recent from North America, using 2D geometric morphometrics to determine the morphological differences among them. Our results show that these three populations were morphologically distinct. The Pleistocene coyotes had an overall robust mandible with an increased shearing arcade and a decreased grinding arcade, adapted for carnivory and killing larger prey; whereas the modern populations show a gracile morphology with a tendency toward omnivory or grinding. The earliest Holocene populations are intermediate in morphology and smallest in size. These findings indicate that a niche shift occurred in coyotes at the Pleistocene/Holocene boundary – from a hunter of large prey to a small prey/more omnivorous animal. Species interactions between Canis were the most likely cause of this transition. This study shows that the Pleistocene extinction event affected species that did not go extinct as well as those that did.

## Introduction

Coyotes (*Canis latrans*) are an important species for their ability to co-exist with humans in urban and suburban areas [1]–[3] where they provide ecosystem services such as control of populations of deer, rodents, and other pests; but also present challenges, such as spreading diseases to domestic animals and preying on pets [3], [4]. Because of the extirpation of larger carnivores such as bears, wolves, and mountain lions, coyotes are the current apex predator in many ecosystems in North America [5]. Coyotes are also a behaviorally labile species with the ability to change activity patterns and ecological niche depending upon their circumstances. Examples include changing pack size and prey preferences depending on whether competitors/predators, such as gray wolves, are present or absent [6]–[8].

Extant coyote subspecies in North America are also highly mobile, colonizing areas at a rapid rate [9]–[11]. This rapid colonization has led to most extant coyote subspecies being somewhat morphologically and genetically homogenous, which complicates subspecies distinctions [9], [10], [12].

As a labile species, coyotes did not always fill the same ecological niches that they fill today. The end of the Pleistocene epoch (circa 11,500 years ago) ushered in the demise of the large mammalian megafauna that roamed North America for millions of years, but also affected the species that did not go extinct, such as coyotes. Meachen and Samuels [12] examined the postcrania of Pleistocene and Holocene coyotes and found that late Pleistocene coyotes from western North America (*Canis latrans orcutti* ≈ late Pleistocene) were larger and more robust than Holocene coyotes. Using their results and the results of another paper on sociality in *Smilodon fatalis* [13], they concluded that the Pleistocene coyotes were more gregarious and hunted larger prey than Recent coyotes and that the end-Pleistocene megafaunal extinctions had a large effect on this niche shift.

Other studies have examined the crania of *C. l. orcutti* from Rancho La Brea and concluded that their crania were larger and more robust than modern coyotes, with shorter rostra and broader carnassial teeth for meat processing [14], [15]. However, these earlier cranial papers did not statistically test the changes that occurred in coyotes, when these changes may have occurred or what the ecological consequences of these changes may have been. In conjunction with the previous work on coyote postcrania, we explore the morphological changes that occurred in coyote crania at the Pleistocene/Holocene boundary that have shaped the roles that coyotes fill today.

Here, we examine the mandibles of seven subspecies of extant coyotes and compare them to the mandibles of coyotes from late Pleistocene Rancho La Brea and early Holocene Rancho La Brea to examine ecological differences in feeding adaptations between these three groups and when any ecological changes may have occurred. The mandible is a good indicator of feeding adaptations because it can be modeled as a two dimensional structure and contains functional information such as potential resistance to chewing forces [16] and relative proportions of grinding versus shearing dentition, which can indicate diet, or in the case of carnivores, prey killing preferences and hunting strategies [17]–[19].

## Materials and Methods

We sampled 76 coyote mandibles from seven extant subspecies from the Field Museum of Natural History (FMNH) including: *C. l. frustor*, *C.l. latrans*, *C.l. lestes*, *C.l. mearnsi*, *C.l. ochropus*, *C.l. texensis*, and *C.l. thamnos*. We also sampled 84 coyote mandibles from the Page Museum (LACMHC) from the Rancho La Brea tar pits (see Table 1 for specimens used). No permits were required for the described study, as no field work was performed to collect these data. Eighteen of these specimens belong to pit 10 at Rancho La Brea. Although some avian specimens from pit 10 are dated as Pleistocene, preliminary data on coyote material suggests that they were indeed early Holocene in age (B. Fuller and J. Southon, personal communication as a continuation of [20]), approximately 8–10 Ka. The remaining 66 mandibles were from Pleistocene pits that range in age from approximately 13–29 Ka [21], including the following pits: 91 (≈29.1 thousand years before present (Kybp)), 16 (≈26.4 Kybp), 3 (≈18.5 Kybp), 13 (≈16.2 Kybp), 4 (≈14.5 Kybp), and 61/67 (≈13 Kybp). Each pit date is a rough estimate rather than a distinct age due to an uncertain window of deposition and a lack of radiocarbon dates.

**Table 1:** Specimen numbers used in this analysis.

Museum	Specimen number	Subspecies	Locality
FMNH	77208	frustror	USA; Arkansas; Miller Co.
FMNH	53694	frustror	USA; Oklahoma; Comanche Co.
FMNH	77209	frustror	USA; Arkansas; Miller Co.
FMNH	135222	frustror	USA; Kansas; Leavenworth Co.
FMNH	53695	frustror	USA; Oklahoma; Comanche Co.
FMNH	13246	mearnsi	USA; California; Tulare Co.
FMNH	13248	mearnsi	USA; California; Tulare Co.
FMNH	13247	mearnsi	USA; California; Tulare Co.
FMNH	53755	mearnsi	USA; California; San Bernardino Co., Yermo
FMNH	13249	mearnsi	USA; California; Inyo Co, Big Cottonwood meadow
FMNH	13251	mearnsi	USA; California; Los Angeles Co, Neenach
FMNH	53705	mearnsi	USA; Arizona; Pima Co.
FMNH	53706	mearnsi	USA; Arizona; Pima Co.
FMNH	53707	mearnsi	USA; Arizona; Pima Co.
FMNH	52860	mearnsi	USA; Arizona; Pima Co.
FMNH	135197	lestes	USA; Wyoming; Sweetwater Barrel Springs
FMNH	135199	lestes	USA; Wyoming; Sweetwater Barrel Springs
FMNH	135201	lestes	USA; Wyoming; Sweetwater Barrel Springs
FMNH	135198	lestes	USA; Wyoming; Sweetwater Barrel Springs
FMNH	105034	lestes	USA; Wyoming, Natrona Co.
FMNH	135200	lestes	USA: Wyoming; Sweetwater, Salazar Butte Quadrangle
FMNH	145970	lestes	USA; Wyoming, Sweetwater Co.
FMNH	160125	lestes	USA; Wyoming, Sweetwater Co.
FMNH	156709	lestes	USA; Wyoming, Sweetwater Co.
FMNH	160124	lestes	USA; Wyoming, Sweetwater Co.
FMNH	145971	lestes	USA; Wyoming, Sweetwater Co.
FMNH	18985	lestes	USA; Colorado, Boulder Co.
FMNH	52901	lestes	USA; Colorado, Mesa Co.
FMNH	18986	lestes	USA; California, Tulare Co.
FMNH	52902	lestes	USA; Colorado, Garfield Co.
FMNH	81499	lestes	USA; California, Tulare Co.
FMNH	20389	lestes	USA; Montana, Jefferson Co.
FMNH	25166	lestes	USA; Idaho, Custer Co., Salmon river
FMNH	20388	lestes	USA; Montana, Jefferson Co.
FMNH	52900	lestes	USA; Colorado, Garfield Co.
FMNH	42765	latrans	USA; South Dakota, Pennington Co.
FMNH	42766	latrans	USA; South Dakota, Pennington Co.
FMNH	42747	latrans	USA; South Dakota, Pennington Co.
FMNH	42764	latrans	USA; South Dakota, Pennington Co.
FMNH	42768	latrans	USA; South Dakota, Pennington Co.
FMNH	42769	latrans	USA; South Dakota, Pennington Co.
FMNH	42748	latrans	USA; South Dakota, Pennington Co.
FMNH	42767	latrans	USA; South Dakota, Pennington Co.
FMNH	13250	ochropus	USA; California, Kern Co,
FMNH	81498	ochropus	USA; California, Los Angeles Co.
FMNH	81495	ochropus	USA; California, Los Angeles Co.
FMNH	81497	ochropus	USA; California, Los Angeles Co.
FMNH	16019	ochropus	USA; California, Mendocino Co.
FMNH	81496	ochropus	USA; California; Los Angeles Co, Alhambra
FMNH	53053	texensis	USA; Texas, Nueces Co, Corpus Christi
FMNH	57504	texensis	USA; Texas, Howard Co
FMNH	83482	texensis	USA; Texas, Brewster Co
FMNH	83481	texensis	USA; Texas, Brewster Co
FMNH	53052	texensis	USA; Texas, Nueces Co, Corpus Christi
FMNH	53051	texensis	USA; Texas; Nueces Co, Corpus Christi
FMNH	154637	thamnos	USA; Illinois, Cook Co. O'Hare
FMNH	126805	thamnos	USA; Illinois, Cook Co.
FMNH	167044	thamnos	USA; Illinois, Cook Co.
FMNH	167068	thamnos	USA; Illinois, Du Page Co. Oak Brook
FMNH	167069	thamnos	USA; Illinois, Du Page Co. Oak Brook
FMNH	172552	thamnos	USA; Illinois, Kane Co.
FMNH	175313	thamnos	USA; Illinois, Du Page Co. Oak Brook
FMNH	196143	thamnos	USA; Illinois, Du Page Co. Hanover Park
FMNH	167043	thamnos	USA; Illinois, Will Co.
FMNH	23946	thamnos	USA; Illinois, Lake Co, Camp Logan
FMNH	129292	thamnos	USA; Illinois, Franklin Co.
FMNH	178025	thamnos	USA; Illinois, Douglas Co.
FMNH	13163	thamnos	USA; Minnesota, Nicollet Co.
FMNH	43961	thamnos	USA; Michigan, Marquette Co.
FMNH	24379	thamnos	USA; Wisconsin, Kenosha
FMNH	129293	thamnos	USA; Wisconsin, Oneida Co.
FMNH	19682	thamnos	USA; Wisconsin, Marinette Co.
FMNH	29513	thamnos	USA; Indiana, St. Joseph Co.
FMNH	154646	thamnos	USA; Wisconsin; Onieda Co.
FMNH	150782	thamnos	USA; Wisconsin, Douglas Co. Brule
LACMHC	HC 6171	orcutti	Pit 3
LACMHC	6172	orcutti	Pit 3
LACMHC	6170	orcutti	Pit 3
LACMHC	6180	orcutti	Pit 3
LACMHC	3201-L-8	orcutti	Pit 3
LACMHC	56861	orcutti	Pit 3
LACMHC	56862	orcutti	Pit 3
LACMHC	57404	orcutti	Pit 4
LACMHC	56915	orcutti	Pit 4
LACMHC	56916	orcutti	Pit 4
LACMHC	6187	orcutti	Pit 4
LACMHC	56931	orcutti	Pit 4
LACMHC	56918	orcutti	Pit 4
LACMHC	56919	orcutti	Pit 4
LACMHC	6186	orcutti	Pit 4
LACMHC	6252	orcutti	Pit 4
LACMHC	56920	orcutti	Pit 4
LACMHC	56921	orcutti	Pit 4
LACMHC	56922	orcutti	Pit 4
LACMHC	HC 6219	orcutti	Pit 10
LACMHC	6220	orcutti	Pit 10
LACMHC	6221	orcutti	Pit 10
LACMHC	6222	orcutti	Pit 10
LACMHC	6223	orcutti	Pit 10
LACMHC	6224	orcutti	Pit 10
LACMHC	6225	orcutti	Pit 10
LACMHC	6226	orcutti	Pit 10
LACMHC	6227	orcutti	Pit 10
LACMHC	6228	orcutti	Pit 10
LACMHC	6229	orcutti	Pit 10
LACMHC	57056	orcutti	Pit 10
LACMHC	57059	orcutti	Pit 10
LACMHC	57061	orcutti	Pit 10
LACMHC	57062	orcutti	Pit 10
LACMHC	57063	orcutti	Pit 10
LACMHC	57064	orcutti	Pit 10
LACMHC	57066	orcutti	Pit 10
LACMHC	57142	orcutti	Pit 13
LACMHC	HC 6210	orcutti	Pit 13
LACMHC	3201-R-5	orcutti	Pit 13
LACMHC	57149	orcutti	Pit 13
LACMHC	57147	orcutti	Pit 13
LACMHC	57151	orcutti	Pit 13
LACMHC	57146	orcutti	Pit 13
LACMHC	57152	orcutti	Pit 13
LACMHC	HC 6213	orcutti	Pit 16
LACMHC	57250	orcutti	Pit 16
LACMHC	57251	orcutti	Pit 16
LACMHC	6255	orcutti	Pit 16
LACMHC	57253	orcutti	Pit 16
LACMHC	57256	orcutti	Pit 16
LACMHC	6254	orcutti	Pit 16
LACMHC	6211	orcutti	Pit 16
LACMHC	57257	orcutti	Pit 16
LACMHC	57259	orcutti	Pit 16
LACMHC	57262	orcutti	Pit 16
LACMHC	HC 6183	orcutti	Pit 61
LACMHC	6192	orcutti	Pit 61
LACMHC	6191	orcutti	Pit 61
LACMHC	6185	orcutti	Pit 61
LACMHC	6175	orcutti	Pit 61
LACMHC	6174	orcutti	Pit 61
LACMHC	3201-R-2	orcutti	Pit 61
LACMHC	57349	orcutti	Pit 61
LACMHC	57350	orcutti	Pit 61
LACMHC	6184	orcutti	Pit 61
LACMHC	57376	orcutti	Pit 67
LACMHC	6199	orcutti	Pit 67
LACMHC	6173	orcutti	Pit 67
LACMHC	6197	orcutti	Pit 67
LACMHC	6200	orcutti	Pit 67
LACMHC	57384	orcutti	Pit 67
LACMHC	6201	orcutti	Pit 67
LACMHC	57378	orcutti	Pit 67
LACMHC	57379	orcutti	Pit 67
LACMHC	57380	orcutti	Pit 67
LACMHC	57381	orcutti	Pit 67
LACMHC	57383	orcutti	Pit 67
LACMHC	57403	orcutti	Pit 67
LACMHC	39573	orcutti	Pit 91
LACMHC	24001	orcutti	Pit 91
LACMHC	13139	orcutti	Pit 91
LACMHC	22036	orcutti	Pit 91
LACMHC	31790	orcutti	Pit 91

Museum legend: FMNH, Field Museum, Chicago, IL; LACMHC, Rancho La Brea, Page Museum Hancock collection, Los Angeles, CA.

We analyzed *C. latrans* mandibular morphology using 2D geometric morphometrics (GM). Mandibular morphology captures many attributes of prey-killing and feeding style in carnivores [16], [22], and mandible fossils are numerous at RLB. Mandibles were digitized from digital photographs of the labial view of hemi-mandibles of *C. latrans.* Our photographing procedure followed published protocols as in [23]. For further discussion of the analysis of 2D representations of 3D structures see Zelditch et al. [24].

**Figure 1:**
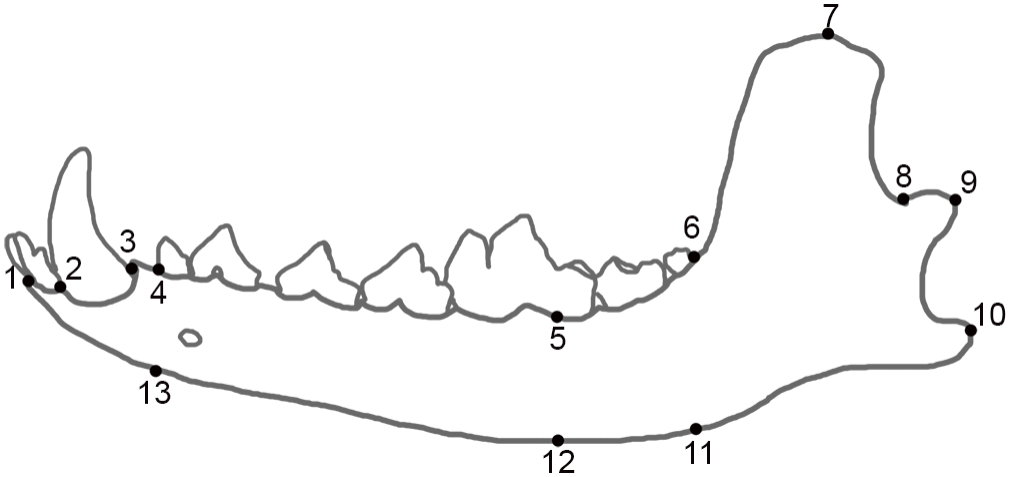
Coyote mandible landmarks used in this study. Also see table 2 for descriptions.

We digitized 13 landmarks on the labial view of each mandible in tpsDig2 (version 2.17) [25]. Landmark points were chosen to represent functional shape changes which may indicate response to feeding stresses and diet (Table 2; Fig. 1). Scalar data was collected by including the scale bar in every specimen photo and using the ‘measure’ tool in tpsDig2 and estimated using the centroid size computed from the landmark data. All sets of landmark coordinates were then aligned using a least-squares Procrustes average configuration of landmarks and the x, y-coordinates were used to obtain a consensus configuration. We generated partial warp scores (localized shape differences) by comparing individual landmarks to the mean configuration [24]. Mandible size and by-proxy overall size [26] were measured using centroid size, the scaling component of the Procrustes superimposition being a robust isometric size estimator [27]. A TPS file of all of our coyote mandibles can be downloaded on Dryad (www.datadryad.org), doi:10.5061/dryad.vn413.

**Table 2:** Landmarks used in this study.

Landmark	Description
1	Anterior edge of mandible, before the incisors.
2	Anterior edge of the canine tooth at the tooth/mandible junction.
3	Posterior edge of the canine tooth at the tooth/mandible junction.
4	Anterior edge of the p1 at the tooth/mandible junction.
5	Point where the talonid basin (grinding surface) begins at the tooth/mandible junction, can be estimated at roughly 2/3 the length of the m1 (carnassial).
6	Posterior edge of the m3 or alveolus at the tooth/mandible junction.
7	Top-most point of the coronoid process
8	Basin of the mandibular notch, used in conjunction with coronoid process to measure coronoid height.
9	Posterior most point of the mandible at the condyloid process.
10	Tip of the angular process
11	Bottom edge of mandible directly below landmark 6, measured with a straight edge
12	Bottom edge of mandible directly below landmark 5, measured with a straight edge
13	Bottom edge of mandible directly below landmark 4, measured with a straight edge

Also see Fig. 1 for graphical representation.

A principal component analysis (PCA) run on the covariance matrix in pcagen7_14a [28] was then used to explore the distribution of mandibular shapes among the samples [24]. Using the PC axes as new variables describing shape variance in the data, we tested the hypotheses of equivalent shape means among pits by running multivariate analyses of variance (MANOVAs) on the resulting PC axes using Scheffé’s *post hoc* procedure for equal variance and Tamhane’s *post hoc* procedure for unequal variances in SPSS 22 [29]. Additionally, we ran a homogeneity of variance test to determine if variances were equal or unequal. A qualitative evaluation of the average shape among pits is accomplished using an animation that shows the movement of each landmark configuration’s mean shape from one pit to another. Arranging the pits in temporal sequence reveals the coyote jaw shape change over geologic time (See S1 Video).

## Results

We ran MANOVAs on the PCs of all possible combinations of coyote groups (modern subspecies, between Pleistocene pits, all groups measured) and found that the comparison between the following three groups were statistically significant: Pleistocene individuals (all pits together – except 10), Pit 10 individuals, and extant individuals (all subspecies taken together) (Table 3), so it is these results that we will focus on for the remainder of the study. No significant differences were found between the PCs of extant coyote subspecies, suggesting that extant coyote jaws show few morphological differences over their geographic range in North America. For the Pleistocene Rancho La Brean coyotes, pits 3 and 61/67 were significantly different on PC 4 (*p* = 0.032), but no other statistically significant differences were found between Pleistocene pits.

**Table 3:** *p*-values for MANOVAs on the principal component scores and centroid size (CS).

Variable	Comparison groups	*p*-value
PC1	Modern vs Pleistocene	**<0.001**
	Modern vs Pit 10	**<0.001**
	Pit 10 vs Pleistocene	0.179
PC2	Modern vs Pleistocene	**<0.001**
	Modern vs Pit 10	**0.007**
	Pit 10 vs Pleistocene	**0.004**
PC3	Modern vs Pleistocene	0.208
	Modern vs Pit 10	**<0.001**
	Pit 10 vs Pleistocene	**0.008**
CS	Modern vs Pleistocene	0.429
	Modern vs Pit 10	**0.006**
	Pit 10 vs Pleistocene	**0.005**

Values in bold indicated significance at the α = 0.05 level.

When we compared modern coyotes (all 7 subspecies together in one group), Pit 10 early Holocene coyotes and Pleistocene RLB coyotes (all Pleistocene pits), our principal components analysis yielded 20 principal components that explained 100% of the variance, however, only the first 3 PCs showed meaningful differences between the three groups. So we chose to focus only on the first 3 PC axes, plus centroid size (CS) for this statistical comparison.

**Figure 2:**
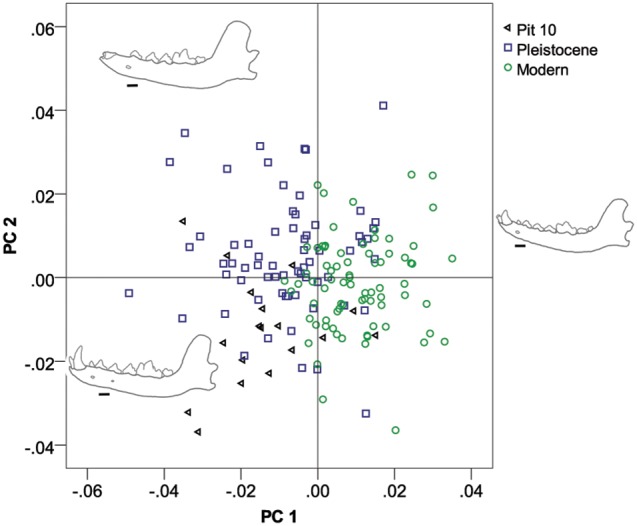
Plot of PC1 versus PC2 for the geometric morphometric Procrustes coordinates for modern coyote, pit 10 coyote, and Pleistocene coyote mandibles.

**Figure 3:**
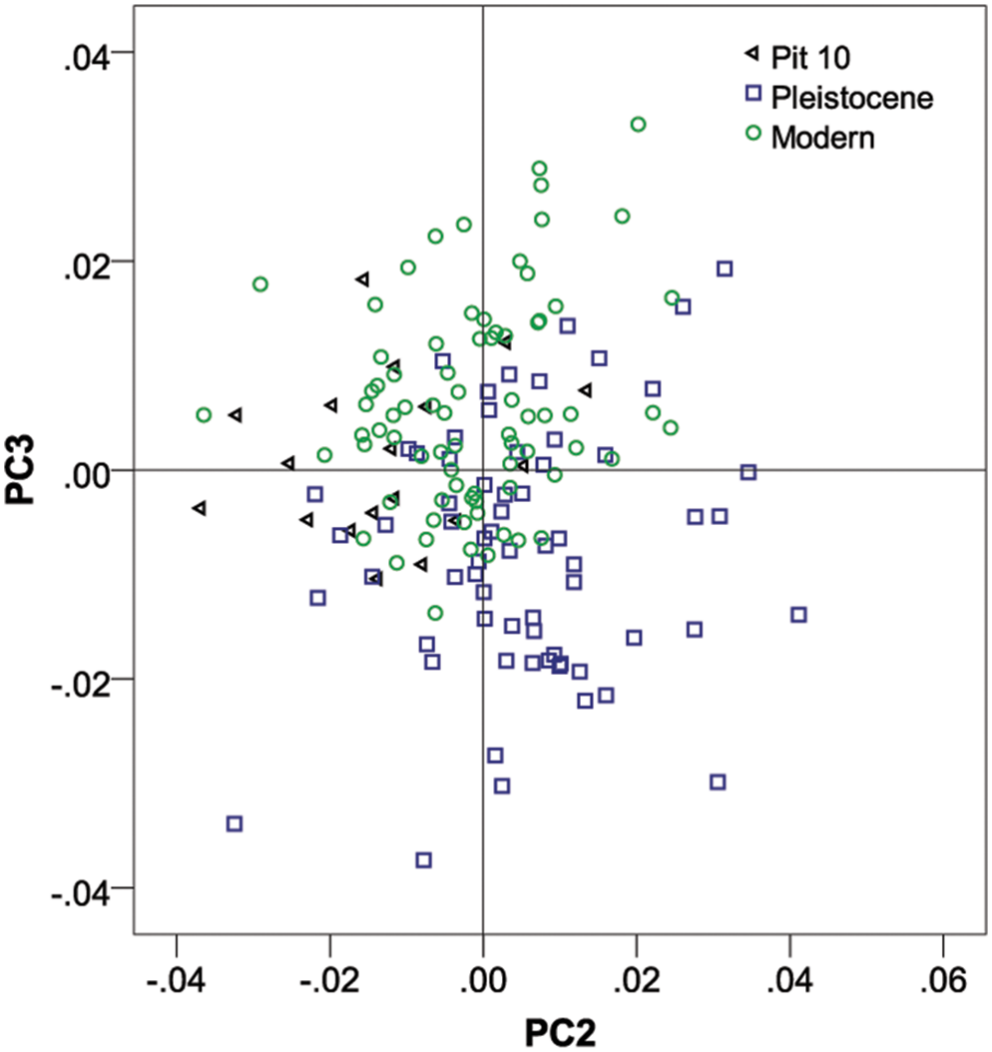
Plot of PC2 versus PC3 for the geometric morphometric Procrustes coordinates for modern coyote, pit 10 coyote, and Pleistocene coyote mandibles.

We found a striking pattern of shape variation between these three coyote groups. On PC1 (22% variance explained), Pit 10 coyotes grouped together with Pleistocene coyotes, with negative values (Fig. 2). The Holocene/Pleistocene Rancho La Brea group had dorso-ventrally deeper mandibles under the pre-molar (shearing) arcade, shallower mandibles under the molar (grinding) arcade, a relatively longer shearing arcade and a relatively short grinding arcade and slightly truncated coronoid processes; whereas all modern coyotes showed the opposite pattern with positive values on PC1– shallow mandibles under the premolars and deeper mandibles under the molars, a relatively short premolar arcade and a relatively longer molar arcade, and a heightened coronoid process. On PC2 (16% variance explained), pit 10 coyotes were significantly different from modern and Pleistocene groups – although the two other groups were not different from each other. Pit 10 coyotes had negative scores with relatively larger canines. Pleistocene coyotes had positive values with smaller canines, while modern coyotes did not differ from the consensus shape on PC2 (Figs. 2 & 3). Due to the possible negative allometry of pit 10 coyotes, relative to Pleistocene coyotes on PC2, we ran a reduced major axis regression of PC2 versus log_10_ centroid size and found that Pleistocene coyotes had the highest slopes with Pit 10 coyotes showing a negative allometric trend and Recent coyotes showing distinct negative allometry from the Pleistocene population (Table 4).

**Table 4:** Reduced major axis regression results for PC2 versus log_10_ CS.

Group	RMA intercept	slope	Slope 95% CI	R^2^
Pleistocene	−2.126	0.651	0.49–0.81	0.0528
Pit 10	−1.870	0.572	0.27–0.87	>0.001
Recent	−1.210	0.371	0.29–0.45	0.0920

Along PC3 (13% variance explained), Pit 10 and modern coyotes grouped together and are significantly distinct from the Pleistocene sample (see Table 3 for *p*-values) (Fig. 3). Pleistocene coyotes had negative scores on PC3, with again, shortening of the grinding area arcade, and an antero-ventral movement of the coronoid process. Positive scores on PC3, consistent with modern specimens, were indicative of shallow mandibles and a postero-dorsal movement of the coronoid process. Pit 10 coyotes did not differ significantly from the consensus shape. For centroid size (CS), pit 10 coyotes were the smallest and were significantly smaller than the Pleistocene sample, but not the modern sample. An animation depicting average pit shape change through time in coyote mandibles can be found in the supplementary materials (S1 Video).

## Discussion

Our findings mirror the postcranial findings of Meachen and Samuels [12]. Pleistocene coyotes from Rancho La Brea are larger and more robust, and modern coyotes are the most gracile, suggesting a change in functional use of the mandible. Our findings also reflect the work of Koblmuller et al. [9] and Thurber and Peterson [10] indicating that modern coyote subspecies are relatively homogeneous. Although the individual specimens of a single subspecies cluster together in morphospace, these clusters are broadly overlapping among subspecies and are not distinct.

Pleistocene coyotes have reduced grinding areas, with an expanded shearing arcade, indicating increased carnivory. Notably, the Pleistocene specimens have thickening of the mandibular corpus directly under the carnassial (apex occurring in pit 61/67), reflecting the increased chewing forces that occurred here [16], [22]. This indicates higher feeding stresses in the mandible, and mandibular thickening continues under the m1–m2 junction which may indicate a higher proportion of durophagy, including large bones in the diet. Van Valkenburgh and Hertel [30] also argued for increased bone consumption in the coyotes of Rancho La Brea as they found a significant increase in tooth breakage. An increase in eating large bones would also necessitate a slightly larger gape, accomplished by the shorter coronoid process. Pleistocene specimens also show corpus thickening at the anterior end, under the first few premolars, when compared with Recent specimens. This anterior mandibular corpus thickening is indicative of increased forces on the anterior mandible, reflecting the “leap and bite” strategy employed by canids when hunting larger prey [19], [31].

Our mandibular results suggest that Pleistocene specimens were large, but not significantly larger than modern specimens. This is slightly different than the findings of Meachen and Samuels [12] that found that Pleistocene coyotes were significantly larger than Recent coyotes. Since postcrania are a better predictor of body mass and overall body size than crania in carnivores [32], we will defer to the postcranial results in this case.

Pit 10 coyotes showed an interesting pattern. These specimens were significantly smaller than all others (as were the postcrania – [12]), but they showed morphological traits closer to their Pleistocene precursors rather than the Recent coyotes; while the subspecies that occurs in southern California today, *Canis latrans ochropus* grouped with the other Recent specimens. Pit 10 coyotes retain the deep mandibular corpus proportions of their predecessors but the relative proportions of shearing versus grinding teeth starts to change. In grinding versus shearing, Pit 10 specimens show an intermediate condition between Pleistocene and Recent populations, the same as the consensus shape. This may indicate a shift away from larger, more difficult prey – the dwindling megafauna, to smaller prey like rabbits and rodents. Additionally, PC2 shows a negatively allometric signal through time. This axis reveals larger canines relative to mandibular size in Pit 10 coyotes, while the larger, Pleistocene coyotes have a relatively smaller canine to mandible size ratio. This may reflect a developmental signal being captured in the earliest Holocene. Neoteny in pit 10 coyotes may be the result of truncated growth in the absence of the large quantity of protein that was available in the Pleistocene. This negative trend continues into the Recent coyote populations.

The intermediate condition in the earliest Holocene coyotes, plus mandibular morphology that closely resembles the Pleistocene specimens, but with negative allometry, suggests an adapting population, rather than immigration from elsewhere. However, we cannot test this hypothesis without a DNA analysis.

Recent coyotes have large but gracile mandibles compared with the older populations. They also have a longer grinding arcade and a shorter shearing arcade compared to earlier populations. The mandibular corpus in the Recent coyotes is also relatively shallow, with a slight thickening under the grinding arcade, posterior 1/3 of m1, m2 and m3, which better reflects their diet today – omnivorous with a focus on smaller prey such as rodents and rabbits, but with occasional large prey, such as deer [3], [7], [8], [33], [34].

In a previous paper Meachen and Samuels [12] discuss the interplay between canids in the late Pleistocene and early Holocene. From late Pleistocene fossil records, gray wolves (*Canis lupus*) are rare in Southern California (and in the lower 48 states), but dire wolves (*Canis dirus*) are common [35], [36]. It is likely that when dire wolves went extinct at the end of the Pleistocene a niche opened up in North America and gray wolves moved across from Eurasia to fill this niche. This transition would have had important ecological consequences for coyotes, which would have a new competitor with a smaller overall body size than dire wolves. This size shift in a major competitor may have forced coyotes to get smaller themselves. In fact, antagonistic relationships have plagued gray wolves and coyotes for centuries, with gray wolves actively hunting and exterminating larger coyote individuals [37]–[43]. It is not unreasonable to think that the megafaunal extinction would have changed the balance between coyotes and larger species of *Canis* in North America.

Additionally, Recent coyote populations also do not seem to follow Bergmann’s rule – a positive relationship between latitude and body size [10], [12] (sometimes presented as a negative relationship between climate and body size). The larger size in Pleistocene coyotes seems to be an anomaly when plotted on a graph of temperature and body size in this species, suggesting that biotic interactions rather than climate are directly responsible for the changes in this species [12].

## Conclusions

Here we show, on average, Pleistocene coyotes were experiencing stronger dorso-ventral forces on the mandible during feeding and hunting than living coyotes. This suggests that Pleistocene coyotes were hunting larger prey more frequently and incorporating harder food (such as bone) into their diet. While it is possible that coyotes were scavenging more than hunting, the shape of the thickened anterior corpus suggests that they were also hunting with a higher frequency. This anterior mandibular thickening is also seen in modern canid species that hunt large prey with regularity, due to the repeated “leap and bite” strategy that transmits forces from the prey to the front of the face in canids [19], [31]. Additionally, since coyotes are the third most common fossil at the Rancho La Brea tar pits, they were trapped with high frequency, which was the argument for sociality that Carbone et al. [13] used in their study. The prior study by Meachen and Samuels [12] suggested that in the Pleistocene coyotes were larger, more carnivorous, and traveled in social packs. Here, we concur with that finding, and from the shape of the mandibular corpus we also suggest that coyotes were not mainly scavenging, but actively hunting larger prey. Meachen and Samuels [12] showed that the major environmental changes that occurred at the end of the Pleistocene were the major drivers for this change, including possibly both the extinction (*Canis dirus*) and influx (*C. lupus*) of other predators and the extinction of many possible prey species.

Coyotes have clearly changed since the Pleistocene and in conjunction with the end-Pleistocene extinction events. Present day species interactions between coyotes and gray wolves give us insight into the evolution of the coyote from what it was in the past into what we see today. Extinction events do not just affect the species that go extinct, but also affect many of the species that remain. For coyotes, interactions between closely related competitors are likely the driving force behind major evolutionary changes.

## Supporting Information

S1 Video Animation that shows the movement of each landmark configuration’s mean shape change through time in *Canis latrans* from approximately 38,000 years ago to present.
(AVI)

## References

[1] Dodge WB, Kashian DM (2013) Recent Distribution of Coyotes Across an Urban Landscape in Southeastern Michigan. Journal of Fish and Wildlife Management 4:377–385.

[2] Gehrt SD, Wilson EC, Brown JL, Anchor C (2013) Population Ecology of Free-Roaming Cats and Interference Competition by Coyotes in Urban Parks. Plos One 8:e75718. 24058699 10.1371/journal.pone.0075718 PMC3772906

[3] Gese EM, Morey PS, Gehrt SD (2012) Influence of the urban matrix on space use of coyotes in the Chicago metropolitan area. Journal of Ethology 30:413–425.

[4] Waser NM, Price MV, Blumstein DT, Arozqueta SR, Escobar BDC, et al (2014) Coyotes, deer, and wildflowers: diverse evidence points to a trophic cascade. Naturwissenschaften 101:427–436. 24728614 10.1007/s00114-014-1172-4

[5] Crooks KR, Soule ME (1999) Mesopredator release and avifaunal extinctions in a fragmented system. Nature 400:563–566.

[6] Arjo WM, Pletscher DH (1999) Behavioral responses of coyotes to wolf recolonization in northwestern Montana. Canadian Journal of Zoology 77:1919–1927.

[7] Gese EM, Rongstad OJ, Mytton WR (1988) Relationship between coyote group-size and diet in Southeastern Colorado. Journal of Wildlife Management 52:647–653.

[8] Ripple WJ, Wirsing AJ, Wilmers CC, Letnic M (2013) Widespread mesopredator effects after wolf extirpation. Biological Conservation 160:70–79.

[9] Koblmuller S, Wayne RK, Leonard JA (2012) Impact of Quaternary climatic changes and interspecific competition on the demographic history of a highly mobile generalist carnivore, the coyote. Biology Letters 8:644–647. 22491760 10.1098/rsbl.2012.0162 PMC3391477

[10] Thurber JM, Peterson RO (1991) Changes in body size associated with range expansion in the coyote (*Canis latrans*). Journal of Mammalogy 72:750–755.

[11] Lydeard C, Kennedy ML (1988) Morphologic assessment of recently founded populations of the coyote, *Canis latrans*, in Tennessee. Journal of Mammalogy 69:773–781.

[12] Meachen JA, Samuels JX (2012) Evolution in coyotes (*Canis latrans*) in response to the megafaunal extinctions. Proceedings of the National Academy of Sciences of the United States of America 109:4191–4196. 22371581 10.1073/pnas.1113788109 PMC3306717

[13] Carbone C, Maddox T, Funston PJ, Mills MGL, Grether GF, et al (2009) Parallels between playbacks and Pleistocene tar seeps suggest sociality in an extinct sabretooth cat, *Smilodon* . Biology Letters 5:81–85. 18957359 10.1098/rsbl.2008.0526 PMC2657756

[14] Nowak RM (1979) North American Quaternary *Canis*. University of Kansas Museum of Natural History Monograph: 1–154.

[15] Giles E (1960) Multivariate analysis of Pleistocene and Recent coyotes (*Canis latrans*) from California. University of California Publications in Geological Sciences 36:369–390.

[16] Biknevicius A, Ruff C (1992) The structure of the mandibular corpus and its relationship to feeding behaviors in extant carnivorans. Journal of Zoology, London 228:479–507.

[17] Biknevicius AR, Van Valkenburgh B (1996) Design for killing: Craniodental adaptations of predators. In: Gittlleman JL, editor. Carnivore Behavior, Ecology, and Evolution. Ithaca, NY: Cornell University Press. 393–428.

[18] Friscia AR, Van Valkenburgh B, Biknevicius AR (2007) An ecomorphological analysis of extant small carnivorans. Journal of Zoology 272:82–100.

[19] Van Valkenburgh B, Koepfli K (1993) Cranial and dental adaptations to predation in canids. Symposium of the Zoological Society of London 65:15–37.

[20] Fuller BT, Fahrni SM, Harris JM, Farrell AB, Coltrain JB, et al (2014) Ultrafiltration for asphalt removal from bone collagen for radiocarbon dating and isotopic analysis of Pleistocene fauna at the tar pits of Rancho La Brea, Los Angeles, California. Quaternary Geochronology 22:85–98.

[21] O'Keefe FR, Fet EV, Harris JM (2009) Compilation, calibration, and synthesis of faunal and floral radiocarbon dates, Rancho La Brea, California. Contributions in Science (Natural History Museum of Los Angeles County) 518:1–16.

[22] Biknevicius A, Leigh S (1997) Patterns of growth of the mandibular corpus in spotted hyenas (*Crocuta crocuta*) and cougars (*Puma concolor*). Zoological Journal of the Linnean Society 120:139–161.

[23] Meachen JA, O'Keefe FR, Sadleir RW (2014) Evolution in the sabertooth cat *Smilodon fatalis* in response to Pleistocene climate change. Journal of Evolutionary Biology 27:714–723. 24779050 10.1111/jeb.12340

[24] Zelditch ML, Swiderski DL, Sheets HD, Fink WL (2012) Geometric Morphometrics for Biologists: A Primer. Amsterdam: Elsevier. 488 p.

[25] Rohlf F (2013) tpsDig2. 2.17 ed. SUNY, Stony Brook, NY.

[26] Figueirido B, Pérez-Claros JA, Hunt RM, Palmqvist P (2011) Body mass estimation in Amphicyonid carnivoran mammals: A multiple regression approach from the skull and skeleton. Acta Palaeontologica Polonica 56:225–246.

[27] Bookstein FL (1997) Morphometric Tools for Landmark Data. Cambridge: Cambridge University Press. 456 p.

[28] Sheets HD (2005) PCAGen7, IMP. 7.14 ed. Canisius College, Buffalo, NY.

[29] IBM (2013) IBM SPSS Statistics for Windows. Aramonk, NY.

[30] Van Valkenburgh B, Hertel F (1993) Tough times at La Brea - tooth breakage in large carnivores of the late Pleistocene. Science 261:456–459. 17770024 10.1126/science.261.5120.456

[31] Ewer R (1973) The Carnivores. Ithaca, New York: Cornell University Press.

[32] Van Valkenburgh B (1990) Skeletal and dental predictors of body mass in carnivores. In: Damuth J, MacFadden B, editors. Body size in mammalian paleobiology: estimation and biological implications. Cambridge: Cambridge University Press. 181–206.

[33] Ogle TF (1971) Predator prey relationships between coyotes and white-tailed deer. Northwest Science 45:213–218.

[34] Ozoga JJ, Harger EM (1966) Winter activities and feeding habits of northern Michigan coyotes. Journal of Wildlife Management 30:809–818.

[35] Dundas RG (1999) Quaternary records of the dire wolf, *Canis dirus*, in North and South America. Boreas 28:375–385.

[36] Leonard JA, Vila C, Fox-Dobbs K, Koch PL, Wayne RK, et al (2007) Megafaunal extinctions and the disappearance of a specialized wolf ecomorph. Current Biology 17:1146–1150. 17583509 10.1016/j.cub.2007.05.072

[37] Berger KM, Gese EM (2007) Does interference competition with wolves limit the distribution and abundance of coyotes? Journal of Animal Ecology 76:1075–1085. 17922704 10.1111/j.1365-2656.2007.01287.x

[38] Berger KM, Gese EM, Berger J (2008) Indirect effects and traditional trophic cascades: A test involving wolves, coyotes, and pronghorn. Ecology 89:818–828. 18459344 10.1890/07-0193.1

[39] Merkle JA, Stahler DR, Smith DW (2009) Interference competition between gray wolves and coyotes in Yellowstone National Park. Canadian Journal of Zoology 87:56–63.

[40] Atwood TC, Gese EM (2008) Coyotes and recolonizing wolves: social rank mediates risk-conditional behaviour at ungulate carcasses. Animal Behaviour 75:753–762.

[41] Atwood TC, Gese EM (2010) Importance of resource selection and social behavior to partitioning of hostile space by sympatric canids. Journal of Mammalogy 91:490–499.

[42] Smith DW, Peterson RO, Houston DB (2003) Yellowstone after wolves. Bioscience 53:330–340.

[43] Krefting LW (1969) The rise and fall of the coyote on Isle Royale. Naturalist 20:24–31.

